# Impact of the prehospital Cardiac Arrest Sonographic Assessment (CASA) mini-course on Knowledge and simulation-based diagnostic performance among Thai paramedic students

**DOI:** 10.1186/s12909-026-09334-0

**Published:** 2026-04-30

**Authors:** Welawat Tienpratarn, Chaiyaporn Yuksen, Wijittra Liengswangwong, Puttipong Surakasamsavat, Saowanee Keram, Janisata Lertsakpanich

**Affiliations:** Department of Emergency Medicine, Faculty of Medicine, Ramathibodi Hospital, Mahidol University, 270 Rama VI Road, Thung Phaya Thai, Ratchathewi, Bangkok, 10400 Thailand

**Keywords:** Heart arrest, Prehospital, Ultrasonography, Medical education, Simulation training

## Abstract

**Introduction:**

Ultrasound use during cardiac arrest, particularly the Cardiac Arrest Sonographic Assessment (CASA) protocol, requires structured integration to identify reversible causes while minimizing interruptions in chest compressions. However, structured CASA training is not yet integrated into paramedic education in Thailand. The aim of this study was to evaluate the impact of a focused CASA mini-course on the knowledge and simulation-based diagnostic performance of Thai paramedic students.

**Methods:**

This prospective pre-post study included third-year paramedic students. Participants completed a 90-minute CASA training course consisting of a 30-minute lecture and a 60-minute hands-on ultrasound session. Knowledge was assessed using a 10-item written test before and after the course. Diagnostic performance was evaluated through simulation-based assessments of CASA skills, including probe placement, image acquisition, and diagnostic interpretation. Participant satisfaction and confidence were also measured using a Likert scale.

**Results:**

The mean post-test score significantly improved from 3.88 ± 1.36 to 8.81 ± 1.33 (*p* < 0.001), with a large effect size (Cohen’s d = 2.68). Post-training diagnostic accuracy varied by condition. For core CASA components, accuracy was 75% for cardiac tamponade and 81.25% for sonographic features suggestive of pulmonary embolism. Ancillary EFAST interpretation accuracy was 62.5%. In simulation, 100% achieved correct probe placement, while 68.75% correctly diagnosed the reversible cause. Satisfaction scores were high, with an average confidence rating of 4.50 ± 0.63 and applicability to practice at 4.81 ± 0.40.

**Conclusion:**

The structured CASA mini-course was associated with significant short-term improvements in knowledge and simulation-based diagnostic performance. These findings support the feasibility of integrating structured ultrasound training into paramedic curricula.

**Supplementary Information:**

The online version contains supplementary material available at 10.1186/s12909-026-09334-0.

## Introduction

Point-of-care ultrasound (POCUS) has become an essential tool in emergency medicine, particularly in the management of critically ill patients, including those undergoing advanced cardiac life support (ACLS) during cardiac arrest resuscitation [1]. POCUS has been applied to help identify potentially reversible causes of cardiac arrest and to assess cardiac activity during resuscitation [2]. However, contemporary resuscitation guidelines emphasize that intra-arrest ultrasound should be performed only by skilled operators and must not cause additional or prolonged interruptions in high-quality chest compressions [3, 4]. Prolonged pauses for imaging may adversely affect resuscitation quality and patient outcomes [5–7]. Therefore, effective and time-sensitive ultrasound use during cardiac arrest requires structured training and competency to minimize procedural delays [8].

In out-of-hospital cardiac arrest (OHCA), ultrasound must be rapid and precisely integrated into resuscitation. The Cardiac Arrest Sonographic Assessment (CASA) exam is a structured protocol designed to minimize interruptions while evaluating key reversible causes of cardiac arrest. The three core phases assess for cardiac tamponade, sonographic features suggestive of pulmonary embolism, and cardiac activity during scheduled pulse checks, each lasting less than 10 seconds [8]. Ancillary views, including lung ultrasound for features suggestive of tension pneumothorax and FAST assessment for intra-abdominal free fluid, may be incorporated when clinically indicated but are not core components of the protocol. 

Ultrasound findings may assist clinical decision-making; however, isolated right ventricular dilation should not be used to diagnose pulmonary embolism, and ultrasound findings should not serve as the sole determinant for termination of resuscitative efforts. In all circumstances, adjunct ultrasound assessment must not delay or compromise high-quality cardiopulmonary resuscitation [3, 4].

Despite the inclusion of ACLS in the third-year paramedic students of the Bachelor of Science in Emergency Medical Operations curriculum at the Faculty of Medicine, Ramathibodi Hospital, Mahidol University, formal training in the CASA exam is currently lacking. This gap limits students’ ability to apply ultrasound in cardiac arrest scenarios, particularly in prehospital settings where timely identification of reversible causes is critical.

The CASA protocol provides a structured, time-limited ultrasound approach to assess potentially reversible causes of cardiac arrest [8]. Early identification of these conditions may support resuscitative decision-making in the prehospital setting.

Although Thailand’s EMS system was established in 1980, limited human resources have led to the integration of paramedics into prehospital care since 2010 [9]. While paramedics have been trained under the Thailand Qualifications Framework for Higher Education (TQF: HEd) [10], there is currently no requirement or standardized curriculum for ultrasound use by paramedics [11]. Training programs remain inconsistent, particularly in low- and middle-income countries, and typically range from brief lectures to two-day courses [12].

Nonetheless, short-duration ultrasound training has proven effective across various EMS settings. Waterman et al. [13] reported that Canadian paramedics, most without prior ultrasound experience, achieved 85.6% accuracy in FAST examinations following a one-hour focused session. Similarly, Bhat et al. [14] found that paramedic and EMT students in the United States were able to accurately identify pneumothorax, pericardial effusion, and cardiac standstill after just one hour of basic instruction. In Thailand, a recent pilot study showed that paramedic students could also attain high diagnostic accuracy in EFAST after brief, structured training [15].

However, ultrasound education in Thailand remains limited, and there is currently no structured curriculum or standardized evaluation framework for the CASA exam in paramedic training. While several investigations have evaluated paramedic ultrasound use in real-world OHCA, structured CASA-specific curricula and competency-based evaluation frameworks remain underdeveloped in low- and middle-income EMS systems.

This study evaluates the feasibility and short-term educational impact of implementing a structured CASA training model with objective competency assessment in a resource-limited paramedic education setting.

## Methods

### Study design and setting

This was a prospective pre-post intervention study conducted at the Queen Sirikit Learning and Research Center, Chakri Naruebodindra Medical Institute, Faculty of Medicine, Ramathibodi Hospital, Mahidol University, Samut Prakan, Thailand. This campus serves as the primary training site for paramedic students throughout their four-year undergraduate program.

### Participants

Eligible participants included third-year paramedic students from the Faculty of Medicine, Ramathibodi Hospital, in the academic year 2022, who had completed Advanced Cardiovascular Life Support (ACLS) training and provided informed consent. Students were excluded if they were unable to attend the CASA mini-course or withdrew consent at any stage of the study.

### Recruitment and consent process

Recruitment was conducted via posters and online registration using Google Forms and QR codes. Interested students were contacted by fourth-year co-researchers, who provided detailed study information and obtained informed consent electronically or in person.

### Intervention: the CASA mini-course

Participants attended a structured 90-minute training session titled “Mini Course: Prehospital CASA Exam for Paramedic Students,” delivered within a single academic day outside regular curriculum hours. The course consisted of a 30-minute didactic lecture followed by a 60-minute hands-on session using portable ultrasound machines. Training included proper probe selection and handling, time-limited image acquisition (within 10 s), and sonographic identification of cardiac and lung structures. During the hands-on practice session, student volunteers were used to demonstrate probe positioning and normal anatomical landmarks. These volunteers were not used during the simulation-based competency assessment. The complete course structure, including lectures, hands-on sessions, and simulation testing, is summarized in Supplementary Table 1.

### Data collection and assessment

Data collection was conducted in three phases:

1. Baseline data: Participants completed a demographic and background questionnaire covering age, sex, academic year, EMS-related work experience, number of cardiac arrest cases managed, and prior exposure to ultrasound training or use.
2. Evaluation:
  Knowledge assessment: A 10-item written test on CASA knowledge was administered before and after the training course, including appropriate timing of ultrasound during resuscitation, identification of reversible causes of cardiac arrest, probe selection, diagnostic interpretation of CASA findings, and appropriate treatment decision-making based on CASA findings.
  Simulation-based skill assessment: Participants were assessed on CASA application during simulated prehospital OHCA scenarios using high-fidelity manikins and a simulation ultrasound system (SONOSIM 2, SON-E-100-5002-DLS). To evaluate performance, we adopted the Quality of Ultrasound Imaging and Competence (QUICk) framework, a validated model developed by Ziesmann et al. [16] for ultrasound education. Based on this framework, we developed a structured checklist aligned with the CASA protocol. The assessment focused on four key components: (1) correct probe placement, (2) time-appropriate scanning (within 10 seconds), (3) image adequacy in terms of anatomical clarity and diagnostic relevance, and (4) accurate interpretation of sonographic findings for reversible causes of cardiac arrest, including cardiac tamponade, sonographic features suggestive of pulmonary embolism, pneumothorax, and intra-abdominal free fluid. This structured approach allowed for consistent, competency-based evaluation of learners during simulation testing. Each student was assessed individually while acting as a team leader, with roles rotated within groups. Each student's performance was evaluated in real time by two emergency medicine instructors with expertise in resuscitation and prehospital ultrasound, both certified by the Emergency Medicine College of Thailand in resuscitative procedures using a structured checklist. For every assessment item, both raters reached an immediate consensus at the point of observation.

The written test and simulation assessment checklist were reviewed by two external emergency physicians with experience in ultrasound and prehospital care. Their suggestions were incorporated to improve content clarity and alignment with the CASA protocol.

3. Post-training survey: Participants completed a post-training Likert-based questionnaire. The survey included a retrospective self-assessment component, in which participants rated their perceived knowledge and confidence before the training (retrospective pre-rating) and after the training (post-rating) at the same time point. Responses were recorded on a 5-point scale ranging from strongly disagree (1) to strongly agree (5). This “then–now” approach was used to reduce response shift bias and allow participants to reassess their baseline perception after exposure to the training content.

### Ethical considerations

This study was approved by the Human Research Ethics Committee of the Faculty of Medicine, Ramathibodi Hospital, Mahidol University, Thailand (COA. MURA2022/762), with the approval date on December 15, 2022. All participants provided informed consent prior to participation. Student volunteers who participated during the hands-on practice session (for demonstration of normal anatomy only) provided written informed consent. The simulation-based assessment of cardiac arrest scenarios was conducted exclusively using high-fidelity manikins and ultrasound simulation software. To ensure confidentiality, all participant data were anonymized using unique study identification codes. No identifiable personal information was used in the data analysis or reporting of results.

### Sample size estimation and statistical analysis

The sample size was estimated based on a previous study by Buaprasert et al. [17], which examined the accuracy of ultrasound performance among paramedic students before and after training. The reported improvement in diagnostic accuracy, reflected by a positive likelihood ratio, was used to calculate the required sample size using STATA version 16.1 (StataCorp, College Station, TX, USA). A one-sample proportion test was applied with the following parameters: null hypothesis proportion = 0.188, anticipated post-intervention proportion = 0.465, one-sided alpha = 0.05, and power = 0.80. The calculation yielded a minimum required sample size of 15 participants.

Data were analyzed using STATA version 16.1. Continuous variables were assessed for normality using the Shapiro–Wilk test. Normally distributed paired data were compared using paired t-tests, whereas non-normally distributed variables were analyzed using the Wilcoxon signed-rank test. Effect size for the primary outcome (knowledge score improvement) was calculated using Cohen’s d for paired samples. A two-sided p-value < 0.05 was considered statistically significant.

## Results

A total of 16 third-year paramedic students participated in the study. The mean age was 22.27 ± 3.06 years, and most participants were female (62.5%).

Only one participant had prior formal EMS employment (5 years as an EMT), whereas all participants had supervised EMS clinical exposure during their undergraduate paramedic training. The median number of OHCA cases encountered, either during prior EMT employment or clinical training rotations, was 2 (IQR 1–3) (Table 1).

**Table 1:** General information of participants (n=16)

Baseline characteristics	N (%)
Gender (Female)	10 (62.5)
Age (years)	22.27±3.06
Mean±SD	
Previous degree	
- High school	15 (93.75)
- EMT	1 (6.25)
Formal EMS employment (years)	1 participant had 5 years of prior EMT employment; all others had no formal EMS employment experience.
Total exposure to out-of-hospital cardiac arrest cases (including prior EMT employment and clinical training rotations), Median (IQR)	2, (1, 3)
Experience in learning/teaching ultrasound (times) Median, IQR	1, (1, 2)
Experience performing ultrasound on patients/simulated patients (times) Median, IQR	1, (1, 3)

*Abbreviation*: *SD* Standard Deviation, *EMS* Emergency Medical Services, *EMT* Emergency Medical Technician, *IQR* Interquartile Range

Following the CASA mini-course, participants demonstrated significant improvement in both objective knowledge and self-assessed understanding. The mean pre-test score increased from 3.88 ± 1.36 to 8.81 ± 1.33 post-training, with a mean difference of 4.94 ± 1.84 (*p* < 0.001) and a large effect size (Cohen’s d = 2.68). Participants’ perceived knowledge improved from 2.50 ± 0.82 (retrospective pre-training self-rating) to 4.81 ± 0.40 (post-training rating) (*p* < 0.001) (Table 2).

**Table 2:** Pre- and post-score in the paper-based test and knowledge of participants

Measure	Score (Mean±SD)
Paper-based test (total 10 points)	
Pre-test mean score	3.88±1.36
Post-test mean score	8.81±1.33
Mean difference	4.94±1.84 (p < 0.001)
Cohen’s d (effect size)	2.68
Knowledge of participants’ evaluation (1-5 Likert scale)	
Perceived knowledge(retrospective pre-training rating)	2.50±0.82
Perceived knowledge (post-training rating)	4.81±0.40
Mean difference	2.31 (p < 0.001)

*Abbreviation*: *SD* Standard Deviation

In the post-test analysis, the highest-performing domains included identification of reversible causes using CASA, CASA appropriate timing, and tension pneumothorax treatment (each 100%). In contrast, the lowest performance was observed in interpreting positive EFAST findings (62.5%). No participants were able to correctly interpret ultrasound findings prior to the training. Following the mini-course, diagnostic accuracy varied by condition, with 62.5% for EFAST, 75% for cardiac tamponade, and 81.25% for sonographic features suggestive of pulmonary embolism. Correct probe selection was achieved in 81.25% of participants. Appropriate management decisions were identified in 93.75% of cases with sonographic features suggestive of pulmonary embolism and in 100% of cases involving tension pneumothorax. Management for cardiac tamponade was not included in the structured written assessment (Table 3).

**Table 3:** 10-Question exam analysis (post-test score)

Aspect	Results
Highest performance topics	100%
	- Duration to do CASA
	- Reversible causes diagnosed by CASA
	- Tension pneumothorax treatment
Lowest performance topics	62.5% (EFAST interpretation)
Ultrasound diagnostic interpretation accuracy	62.5-81.25%
	- 62.5% (EFAST positive and view)
	- 75% (cardiac tamponade and view)
	- 81.25% (sonographic features suggestive of pulmonary embolism and view)
Probe selection for CASA exam	81.25%
Plan of management after diagnosis	- Sonographic features suggestive of pulmonary embolism: 93.75%
	- Tension pneumothorax: 100%

*Abbreviation*:* CASA* Cardiac Arrest Sonographic Assessment, *EFAST* Extended Focused Assessment with Sonography for Trauma

Simulation-based assessments using the QUICk criteria showed that all participants (100%) achieved correct probe placement. However, only 62.5% acquired ultrasound images within 10 s, and 56.25% produced adequately optimized images. A total of 68.75% of participants correctly diagnosed the underlying reversible cause of cardiac arrest in the simulation scenario (Table 4).

**Table 4:** QUICk criteria evaluation summary from CASA simulation test (n=16)

QUICk Component	Result N (%)
Image acquisition within 10 seconds	10 (62.50)
Image optimization (adequate view)	9 (56.25)
Correct probe location	16 (100)
Correct diagnosis of reversible cause	11 (68.75)

*Abbreviation*: *QUICk* Quality of Ultrasound Imaging and Competence, *CASA* Cardiac Arrest Sonographic Assessment

Participants reported high levels of satisfaction with the course. Agreement with statements related to confidence in applying CASA averaged 4.50 ± 0.63 on a 5-point Likert scale. Agreement regarding applicability to real practice averaged 4.81 ± 0.40. Satisfaction with instruction and equipment approached the maximum score (mean 5.00), and perceived appropriateness of training duration averaged 4.75 ± 0.58. Detailed item-level responses are presented in Supplementary Table 2.

## Discussion

This study demonstrates significant short-term improvements in knowledge and simulation-based diagnostic performance following a structured CASA mini-course. The findings support the feasibility of integrating structured ultrasound education into paramedic curricula in resource-limited settings. However, the incremental benefit of simulation-enhanced training over lecture-only formats remains uncertain and warrants randomized comparison. As this study did not assess real-world OHCA performance, its contribution lies primarily in the development and objective evaluation of a structured CASA educational framework rather than in clinical outcome assessment.

Consistent with our results, previous studies have shown that even short training interventions can meaningfully improve ultrasound competency. Rooney et al. [18] demonstrated that paramedics with minimal training could acquire adequate cardiac ultrasound images and accurately detect cardiac activity in the field, reinforcing the feasibility of integrating protocols like CASA into prehospital care. Similarly, Ohira et al. [19] found that repeated simulation-based practice significantly improved both the speed and image quality of EFAST exams performed by paramedics, underscoring the value of hands-on repetition for developing technical proficiency.

Objective assessment is critical in ultrasound education. The QUICk score [16] offers a structured and reliable method for evaluating image quality and diagnostic performance, distinguishing novices from experts with high accuracy. The incorporation of QUICk-based assessments in the present CASA training further supports its utility in evaluating competency across probe placement, image acquisition, and interpretation.

The broader literature also emphasizes the variability in ultrasound training across EMS systems. A review by Höhne et al. [17] highlighted the lack of standardized evaluation frameworks and recommended multi-method assessments that address both technical and interpretive skills. Meadley et al. [11] similarly found inconsistencies in training duration and delivery in paramedic programs, though brief, focused instruction, like the CASA mini-course, was frequently associated with gains in learner performance.

In terms of content relevance, Mallary et al. [20] highlighted ultrasound’s diagnostic value in prehospital care, particularly in identifying life-threatening conditions like pneumothorax and hemoperitoneum. These findings align with the CASA protocol, which targets key reversible causes of cardiac arrest (e.g., tamponade, PE, pneumothorax, intra-abdominal bleeding) through a structured, time-sensitive approach. Moreover, evidence from the PECA study [21] showed that focused cardiac ultrasound can be integrated into OHCA management without delaying key resuscitation steps and may guide real-time treatment decisions.

Roche et al. [22] further demonstrated that ultrasound training enhanced paramedics’ clinical decision-making, leading to improved diagnostic accuracy, transport prioritization, and pre-arrival notification. In the Southeast Asian context, Ienghong et al. [23] found that emergency medicine trainees in Laos benefited from a short, hands-on elective covering CASA and EFAST, reporting improved knowledge and high satisfaction with the peer-led teaching model, highlighting regional feasibility and acceptance of the training model used in this study.

Together, these findings validate the CASA mini-course as an effective, context-appropriate educational strategy to enhance diagnostic readiness among paramedic students. While theoretical knowledge can be improved within a short time frame, the practical elements, especially image acquisition within the time constraints of resuscitation, require ongoing practice. As portable ultrasound becomes more accessible in Thai EMS, structured training such as CASA can bridge the gap between knowledge and prehospital clinical application, ultimately improving patient care in cardiac arrest scenarios.

## Limitations

This study has several limitations. First, it was a single-group pre-post study without a comparator group. As participants had minimal prior CASA-specific training, improvements in knowledge and performance were expected following structured instruction. Therefore, the findings reflect short-term educational gain rather than comparative effectiveness of simulation over alternative teaching modalities. Second, the sample size was small (*n* = 16) and limited to third-year paramedic students from a single institution, which may restrict generalizability. Third, assessment was conducted in a simulated environment, which may not fully replicate the complexity and cognitive demands of real out-of-hospital cardiac arrest scenarios. Fourth, no follow-up evaluation was performed to assess long-term retention or clinical translation. Fifth, the use of a retrospective pre–post (“then-now”) Likert design may introduce recall bias and may not fully reflect baseline perceptions prior to training. Finally, although the mini-course included both didactic and hands-on components, the duration of practical exposure was brief and may not reflect optimal training intensity.

## Conclusion

This study demonstrates significant short-term improvements in paramedic students’ knowledge and simulation-based diagnostic performance following a structured, short-duration CASA mini-course. These findings support the feasibility of integrating focused ultrasound training into paramedic curricula, particularly in resource-limited EMS systems. Future studies should compare simulation-based and didactic-only CASA training and assess long-term retention.

## Supplementary Information

Supplementary Material 1.

## Data Availability

The datasets generated during and/or analysed during the current study are available from the corresponding author on reasonable request.

## Abbreviations

ACLS: Advanced Cardiovascular Life Support
CASA: Cardiac Arrest Sonographic Assessment
EFAST: Extended Focused Assessment with Sonography for Trauma
EMS: Emergency Medical Services
EMT: Emergency Medical Technician
FAST: Focused Assessment with Sonography for Trauma
OHCA: Out-of-Hospital Cardiac Arrest
PE: Pulmonary Embolism
PEA: Pulseless Electrical Activity
POCUS: Point-of-Care Ultrasound
QUICk: Quality of Ultrasound Imaging and Competence
SD: Standard Deviation
TQF: HEd – Thailand Qualifications Framework for Higher Education
