# Supplementary material for: Impact of the prehospital Cardiac Arrest Sonographic Assessment (CASA) mini-course on Knowledge and simulation-based diagnostic performance among Thai paramedic students
Source: BMC Med Educ. 2026 Apr 30;26:1003. doi: 10.1186/s12909-026-09334-0 (PMC13281339; doi:10.1186/s12909-026-09334-0)
Supplement: Supplementary file 1 — Supplementary Material 1. [file 12909_2026_9334_MOESM1_ESM.docx]

Supplementary Table 1. Structure of the CASA mini-course and assessment process

| **Activity** | **Content** |
| --- | --- |
| Registration and pre-activity  (15 minutes) | - Complete a pre-test - Provide general information along with prior experience in performing ultrasound |
| CASA lecture and Q&A session  (30 minutes) | - Introduction to CASA and its role in cardiac arrest - Standard CASA views and protocol - Identification of reversible causes (Hs and Ts) - Case scenarios and image interpretation practice |
| Hands-on CASA session  (60 minutes) | - Probe handling and standard imaging views - Practice identifying reversible causes during cardiac arrest |
| Post-activity  (15 minutes) | - Complete a post-test - Provide confidence and satisfaction evaluation form |
| CASA simulation test  (120 minutes) | - Team-based simulation with individual performance assessment (4 case scenarios)   1. Tension pneumothorax   2. Cardiac tamponade   3. Sonographic features suggestive of pulmonary embolism   4. Intra-abdominal free fluid due to ruptured ectopic pregnancy |

Abbreviation: CASA, Cardiac Arrest Sonographic Assessment

The structured CASA educational intervention consisted of a 90-minute session (a 30-minute lecture and a 60-minute hands-on practice). Pre/post-testing and simulation assessments were conducted as part of the study evaluation and were not included in the instructional duration.

Supplementary Table 2. Participant evaluation (confidence, applicability, and satisfaction) after the CASA mini-course (5-point Likert scale; 1 = strongly disagree, 5 = strongly agree) (n=16)

| **Participant evaluation** | **Score (Mean±SD)** |
| --- | --- |
| Confidence in CASA use | 4.50 ± 0.63 |
| Application to practice | 4.81 ± 0.40 |
| Instructor and teaching satisfaction | 5.00 |
| Training time suitability | 4.75 ± 0.58 |
| Equipment readiness | 5.00 |
| Test appropriateness (paper and simulation) | 4.94 ± 0.25 |

Abbreviation: CASA, Cardiac Arrest Sonographic Assessment
